# De novo assembly of transcriptome dataset from leaves of *Dryobalanops aromatica* (Syn. *Dryobalanops sumatrensis*) seedlings grown in two contrasting potting media

**DOI:** 10.1186/s13104-020-05251-7

**Published:** 2020-08-28

**Authors:** Iskandar Zulkarnaen Siregar, Fifi Gus Dwiyanti, Ulfah Juniarti Siregar, Deden Derajat Matra

**Affiliations:** 1 Department of Silviculture, Faculty of Forestry and Environment, IPB University (Bogor Agricultural University), Bogor, Indonesia; 2 Department of Agronomy and Horticulture, Faculty of Agriculture, IPB University (Bogor Agricultural University), Bogor, Indonesia

**Keywords:** Adaptive genetic variation, *Dryobalanops aromatica*, *Dryonalanops sumatrensis*, Peat swamp, Transcriptome

## Abstract

**Objectives:**

Efforts to restore tropical peat swamp forests in Indonesia face huge challenges of potential failures due to socio-economic factors and ecological dynamics attributed to lack of knowledge on the adaptive mechanisms of potential tree species such as Kapur (*Dryobalanops aromatica* C.F.Gaertn Syn. *Dryobalanops sumatrensis* J.F. Gmelin A.J.G.H Kostermans). This species is a multi-purpose tree that, commonly grows in mineral soils, but also in peat swamp as previously reported, which raised a fundamental question regarding the molecular mechanism of this adaptation. Therefore, a dataset was created aiming to detect candidates of adaptive genes in *D. aromatica* seedlings, cultivated in two contrasting potting media, namely mineral soil and peat media, based on RNA Sequencing Transcriptome Analysis.

**Data description:**

The RNA transcriptome data of *D. aromatica’*s seedlings derived from young leaves of three one-year-old seedlings, raised in each dry mineral soil media and peat media, were generated by using Illumina HiSeq 4000 platform in NovogenAIT, Singapore. The acquired data, as the first transcriptome dataset for *D. aromatica,* is of a great importance in understanding molecular mechanism and responses of the involved genes of *D. aromatica* to the contrasting, growing potting media conditions that could also be useful to generate molecular markers.

## Objective

The past genetic research on *Dryobalanops aromatica* focused on pattern of genetic variation and population structure in North-eastern Borneo, Sumatera, and the Malay Peninsula using nuclear microsatellite markers [1]. The investigated ecosystem types for all populations were from mineral soil forest types, in which *D. aromatica* could be found abundantly on deep, humid, yellow, sandy soils with a propensity for ridges [2]. However, it was recently discovered that this species also grows in peat swamp forest, as found in Singkil Wildlife Reserve (Suaka Margasatwa Singkil), Aceh, Sumatera. According to this finding, the former investigation was then concentrated on how to understand life-history characteristics such as comparing shoot cuttings ability of *D. aromatica* in peat and coco peat media [3]. In addition, due to lack of in-depth investigation of adaptive genetic variation of this species grown in mineral soil and peat media, an experiment was carried out through RNA sequencing (RNA-Seq) transcriptome analysis. Studies on adaptive genetic analysis using RNA-Seq in tropical forest trees have previously been reported, such as research on *Shorea balangeran* adaptation grown in mineral and peat potting media [4] and gall-rust infected and uninfected trees of *Falcataria moluccana* [5]. Considering potential application of transcriptome analysis on forest trees, similar research was also conducted on *D. aromatica*. Objective of the research was to detect candidates of adaptive genes in *D. aromatica* seedlings, grown in two contrasting potting media, namely mineral soil and peat media. The findings were expected to provide more accurate information on molecular adaptive mechanism for practical use to support rehabilitation and conservation of degraded peat swamp forests in Indonesia. Results of the study are presented in Table 1.

**Table 1:** Overview of data files/data sets

Label	Name of data file/data set	File types (file extension)	Data repository and identifier (DOI or accession number)
Data file 1	Transcriptome assembly contigs	Fasta file (.fasta)	Figshare 10.6084/m9.figshare.12326177.v5 [19]
Data file 2	Summary for alignment of clean reads to reference transcriptome	Document file (.docx)	Figshare 10.6084/m9.figshare.12326177.v5
Data file 3	Functional annotation from non-redundant nucleotide NCBI	BLAST output in txt/-outfmt 6 option (.txt)	Figshare 10.6084/m9.figshare.12326177.v5
Data file 4	Functional annotation from non-redundant protein NCBI	BLAST output in XML/-outfmt 5 option (.xml)	Figshare 10.6084/m9.figshare.12326177.v5
Data file 5	Functional annotation from protein sequence database of SwissProt	BLAST output in XML/-outfmt 5 option (.xml)	Figshare 10.6084/m9.figshare.12326177.v5
Data file 6	Functional annotation from protein sequence database of TrEMBL	BLAST output in txt/-outfmt 6 option (.txt)	Figshare 10.6084/m9.figshare.12326177.v5
Data file 7	Statistics related to contig length distribution and the Blast results: e-value distribution, contig similarity distribution, top-hit species distribution	PNG files in compressed file (.zip)	Figshare 10.6084/m9.figshare.12326177.v5
Data file 8	Functional annotation from Complete TREP nucleotide database	BLAST output in txt/-outfmt 6 option (.txt)	Figshare 10.6084/m9.figshare.12326177.v5
Data file 9	Functional annotation from Hypothetical TREP protein database	BLAST output in txt/-outfmt 6 option (.txt)	Figshare 10.6084/m9.figshare.12326177.v5
Data file 10	Assessing transcriptome assembly and annotation completeness with single-copy orthologs by BUSCO	BUSCO output in txt (.txt)	Figshare 10.6084/m9.figshare.12326177.v5
Data file 11	Gene Ontology and KEGG analysis	Blast2GO file (.b2g)	Figshare 10.6084/m9.figshare.12326177.v5
Data file 12	Open Reading Frames (ORFs) prediction	Fasta File (.fasta)	Figshare 10.6084/m9.figshare.12326177.v5
Data file 13	Results of microsatellite region finding	Misa file (.txt)	Figshare 10.6084/m9.figshare.12326177.v5
Data set 1	Raw RNA‐seq. reads and assembled contigs	Fastq files (.fastq)	DNA Data Base of Japan (https://identifiers.org/insdc.sra:DRP005979 [20]

## Data description

*Dryobalanops aromatica*’s seedlings, collected from Lae Kombih Forest Park, Aceh, Sumatera and transported to greenhouse of Department of Silviculture, IPB University, Bogor, were treated under two contrasting types of potting (diameter 10 cm) fine media, i.e., mineral soil (n = 3 seedlings) and peat (n = 3 seedlings) with regular watering. Peat media was classified as fibric peat, which has pH of 4.0 and 135.32% water content, whereas mineral soil media is classified as clay loam soil which has pH of 5.0 and 32.09% water content. Total RNA from young leaves collected from three one-year-old seedlings cultivated in each mineral soil media and peat media were extracted by using Plant Total RNA Mini Kit (Geneaid Biotech Ltd), following manufacturer’s instructions. The integrity and quantity of extracted-RNA were measured by using NanoDrop ND-1000 spectrophotometer and Agilent 2100 Bioanalyzer.

The RNA sequencing was undertaken using Illumina HiSeq 4000 (Novogene-AIT, Singapore) that produced pre-processing reads, which afterwards became subjects to discard the library adaptors and low-quality reads below Q < 30 (data set 1). The clean reads were de novo assembled by Trinity 2.3.2 [6], and the redundant transcripts were removed using CAP3, cd-hit-est, and corset 1.08, respectively [7–9]. Sequencing the yielded 221 million reads produced total 114,268 contigs. The contigs ranged from 201 to 50,886 base pairs with N50 of 1970 bp (data file 1). To assess the quality of transcriptome reference, clean reads were mapped to reference using Bowtie2 [10] (Data file 2).

The functional annotation of contigs was performed using BLAST + 2.7.1 program against the NCBI nr (data file 3), NCBI nt (data file 4) (downloaded by 6th October 2018 and subjected to Euphyllophyta) and SwissProt (data file 5) and TrEMBL (data file 6) (downloaded by 3rd January 2020) databases with an E-value cutoff of 10^−5^ [11, 12]. Statistics of transcriptome reference were analyzed using Blast2GO 5.2 [13] that produced statistics of length distribution and Blast results with NCBI nr as follows: e-value distribution, contig similarity distribution and top-hit species distribution (data file 7). Functional analysis showed that 80,507 (70.45%) indicated significant matches with NCBI nr as well as 59,353 (51,94%) in the SwissProt database. The transposon sequence analysis was analyzed using BLAST program with TREP database [14] (data file 8, data file 9). Transcriptome reference was assessed using Busco v.3.2 [15] under Maser platform [16] (data file 10). The SwissProt-annotated contigs were used to analyze GO and KEGG pathways using Blast2GO 5.2 (data file 11).

To predict ORFs, the contigs were analyzed using TransDecoder 5.5.0 [17] (data file 12). A total of 84,175 contigs was identified as ORFs with 5′prime partial of 13,430 (15,95%), 3′prime partial of 8574 (10,19%) and complete ORFs type of 57,306 (68,08%). Contigs containing microsatellite were extracted by using the MISA program [18], with minimum repeats such as: 10 for one base, 6 for two bases, and 5 for 3, 4, 5, and 6 bases; and the interruptions between sites of microsatellite were 100 bases. The microsatellite motifs containing contigs were summed up to 39,025 (data file 13).

## Limitations

The seedlings were not collected directly from the field due to the lack of natural regeneration and remarkably lengthy distance. Rather, seedlings were treated in two types of potting media (i.e. mineral and peat) grown in the green house with regular maintenance. Furthermore, RNA extraction samples were obtained from the leaves, only leaving other plant parts to be analyzed for better comparisons due to already established RNA extraction methods for the leaves. The extraction was also carried out solely once during sampling point in order to meet the sufficient replicates.

## Data Availability

The data described in this Data note can be freely and openly accessed on figshare (10.6084/m9.figshare.12326177.v5) and DNA Data Base of Japan (https://identifiers.org/insdc.sra:DRP005979). Please see Table 1 and references list [19, 20] for details and links to the data.

## Abbreviations

RNA-Seq: RNA sequencing transcriptome analysis
nr: Non-redundant protein
nt: Nucleotide sequences
TREP: The TRansposable Elements Platform
GO: Gene Ontology
KEGG: Kyoto Encyclopedia of Genes and Genomes
BP: Biological processes
MF: Molecular function
CC: Cellular component
ORFs: Open reading frames
